# What can we learn from the effects of the COVID-19 pandemic on hospital care for children in Germany?

**DOI:** 10.1093/eurpub/ckac129.333

**Published:** 2022-10-25

**Authors:** N Mauer, F Tille, U Nimptsch, D Panteli

**Affiliations:** 1 Brussels Hub, European Observatory on Health Systems and Policies, Brussels, Belgium; 2 London Hub, European Observatory on Health Systems and Policies, London, UK; 3 Department of Healthcare Management, Technische Universitaet Berlin, Berlin, Germany

## Abstract

The COVID-19 pandemic created substantial disruptions in the delivery of health services around the world. Reductions in hospital admissions have been reported for several conditions in the adult population; less evidence currently exists for children. To what extent such changes reflect a risk for patients due to unmet care needs, or a “correction” of previous overprovision of care has not been thoroughly examined yet. Based on complete national hospital discharge data, we compare the top 30 diagnoses for which children were hospitalised in 2019, 2020 and 2021 in Germany. We also analyse the development of monthly admissions between January 2019 and December 2021 for three tracers of variable urgency and severity. Total admissions were approximately 20% lower in 2020 and 2021 compared to 2019. The composition of the most frequent diagnoses did not change dramatically across years, although changes in rank were observed. The number of admissions for acute lymphoblastic leukaemia (tracer 1) showed a slight increasing trend and a periodicity prima vista unrelated to pandemic factors. Appendicitis admissions (tracer 2) decreased by about 9% in 2020 and a further 8% in 2021, while tonsillectomies/adenoidectomies (tracer 3) decreased by more than 40% in 2020 and a further 30% in 2021; for these tracers, monthly changes are in line with pandemic waves. Observed variations in child hospitalisations reflect the effects of pandemic mitigation measures and/or changes in demand. In Germany, inpatient care for critical conditions appears to have been largely upheld, potentially at the expense of elective treatments. Complementary data on ambulatory care and health outcomes would enable a better understanding of change in healthcare patterns and effects on children's health.

